# Serine/arginine-rich splicing factors: the bridge linking alternative splicing and cancer

**DOI:** 10.7150/ijbs.46751

**Published:** 2020-07-06

**Authors:** Xiang Zheng, Qiu Peng, Lujuan Wang, Xuemei Zhang, Lili Huang, Jia Wang, Zailong Qin

**Affiliations:** 1 Laboratory of Genetics and Metabolism, Maternal and Child Health Hospital of Guangxi Zhuang Autonomous Region; Guangxi Birth Defects Research and Prevention Institute, Nanning, Guangxi, 530003, China.; 2 Department of Pathology, Affiliated Hospital of Guilin Medical University, Guilin, Guangxi, 541001, China.; 3 Department of Immunology, Changzhi Medical College, Changzhi, Shanxi, 046000 China.; 4 Cancer Research Institute, School of Basic Medical Science, Central South University, Changsha, Hunan, 410008, China.

**Keywords:** serine and arginine-rich factors (SRs), SRSF, cancer, alternative splicing, splicing regulation, N6-methyladenosine (m6A), arginine methylation

## Abstract

The serine/arginine-rich splicing factors (SRs) belong to the serine arginine-rich protein family, which plays an extremely important role in the splicing process of precursor RNA. The SRs recognize the splicing elements on precursor RNA, then recruit and assemble spliceosome to promote or inhibit the occurrence of splicing events. In tumors, aberrant expression of SRs causes abnormal splicing of RNA, contributing to proliferation, migration and apoptosis resistance of tumor cells. Here, we reviewed the vital role of SRs in various tumors and discussed the promise of analyzing mRNA alternative splicing events in tumor. Further, we highlight the challenges and discussed the perspectives for the identification of new potential targets for cancer therapy via SRs family members.

## Introduction

Alternative pre-mRNA splicing (AS) is an important gene regulation process that greatly enriches the repertoire of the transcriptome, contributing to transcriptomic and proteomic diversity 1. AS is emerging as a pivotal step that tightly regulated in different tissues, differentiation stages and important cellular pathways in higher eukaryotes 2-4. Growing studies showed that, AS process, which allows production of multiple mRNA variants from each gene and represents an evolutionary advantage for higher eukaryotes, and the alterations of AS contributing to various diseases including growth hormone deficiency, Parkinson's disease and cancer 5. AS is a sophisticated and ubiquitous nuclear process, which is a natural source of cancer-causing errors in gene expression 6. Increased high-throughput sequencing of the genome and transcriptome of cancers has unveiled a variety of means by which pre-mRNA splicing is altered in cancer 2. As AS products are usually consistent with active roles in cancer, the alternative splicing process itself is a potential target for gene therapy.

Pre-mRNA splicing relies on the binding of spliceosome and some RNA-binding proteins (RBPs) to the pre-mRNA cis-acting element, and involves a series of RNA-RNA, RNA-protein, and protein-protein interactions 7-9. However, there are also splicing regulatory elements, especially in the exons, including enhancers or silencers located within introns and exons region of gene (exonic or intronic splicing enhancers (ESE or ISE) or silencers (ISS or ESS)), regulating splicing by combining with the corresponding trans-acting factors 10. As the key recognizers of these elements, serine and arginine-rich splicing factors (SRs) are concentrated in spliceosome and play vital roles in regulation of alternative splicing 11. Extensive evidence has proved that SRs are involved in nearly every step of spliceosome assembly 12. In addition, SRs have also been demonstrated to be associated with genomic stability, mRNA export, mRNA stability and translation 13, 14.

SRs, the important splicing regulators, belong to the serine arginine-rich protein family and canonically consisted of 12 members (SRSF1-12) 15. The first SRs identified were SRSF1 (previously called SF2/ASF) and SRSF2 (previously called SC35) 16. Most SRs are located exclusively in the nucleus, but some SRs (such as SRSF1, SRSF3 and SRSF7) can shuttle between nucleus and cytoplasm 17. Because SRs play a pivotal role in transcription and translation, dysregulation of SRs greatly disrupts both DNA stability and normal protein expression pattern, then leads to abnormal biological function. Recently, it has been widely studied that SRs act as oncoproteins in various tumor types, such as lung, colon, breast, pancreas and leukemia cancer 18-21. In this review, we summarized the function and mechanism of SRs in RNA splicing and their roles in tumor progression. We also discussed the role of SRs as potential therapeutic targets for cancer therapy.

## Structure and Function of SRs

### Structure of SRs

**Figure 1:**
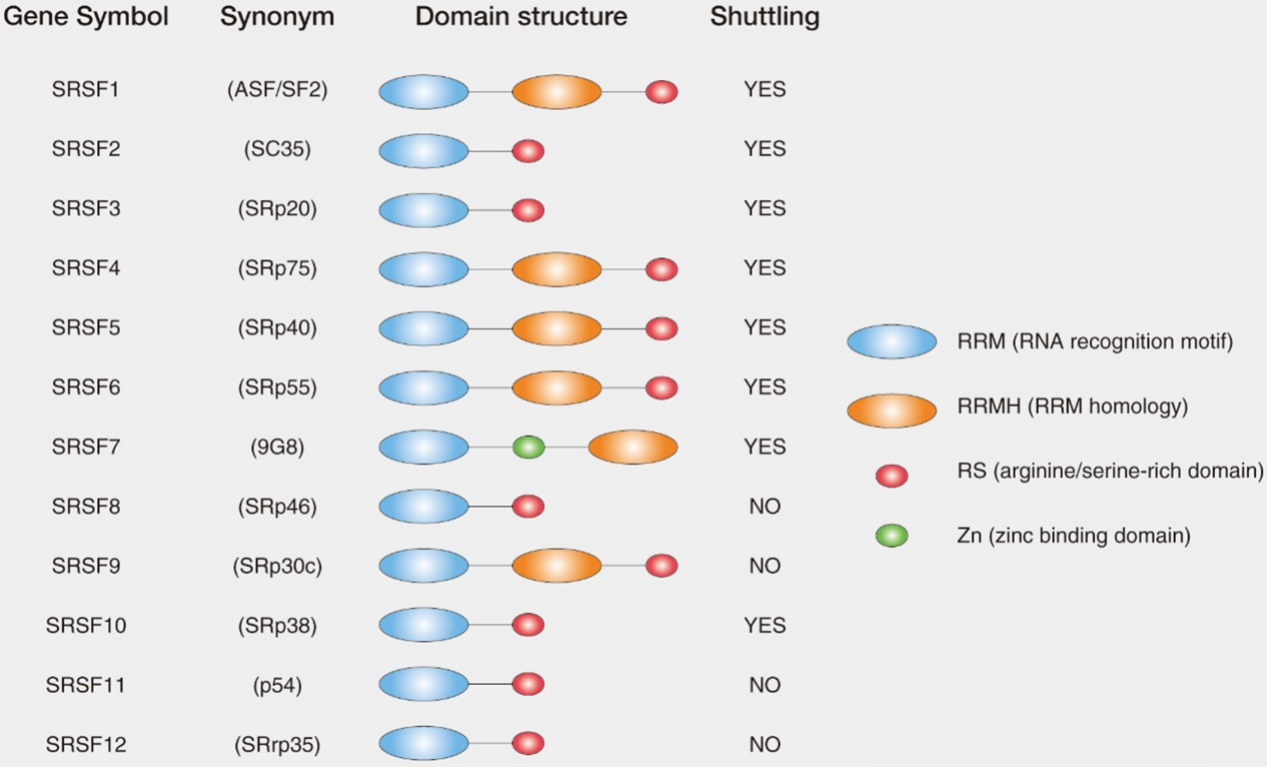
** List and domain structures of SRs**. The domain structure of core SRs consist of RNA recognition motif (RRM), RRM homology (RRMH), arginine/serine-rich domain (RS), zinc binding domain (Zn). Synonym for SRs are also indicated in the parenthesis. Among 12 core SRs, SRSF1, SRSF2, SRSF3, SRSF4, SRSF5, SRSF6, SRSF7 and SRSF10 were reported to shuttle between nucleus and cytoplasm (shuttling SRs), whereas the other SRs have not been shown to have shuttling activity.

SRs are serine/arginine-rich splicing factors which are very conserved in plants and animals 22, 23. The SRs were first discovered in the early 1990s as the essential splicing factors that influenced alternative splicing in Hela cells 24, 25. SRs consist of 12 members in mammalian (SRSF1-12), which have the very similar domains. SRs are characterized by the presence of an N-terminal 1 or 2 RNA recognition domains (RRM domain) and a C-terminal domain enriched with the Arginine (R) and Serine (S) amino acid sequences (RS domain) 26 (Fig. 1). In general, RRM domains recognize RNA and determine the binding of SRs to RNA, while RS domains mediate diverse protein-protein and protein-RNA interactions regulated by phosphorylation 16.

### Function of SRs

#### Function of SRs in alternative pre-mRNA splicing

**Figure 2:**
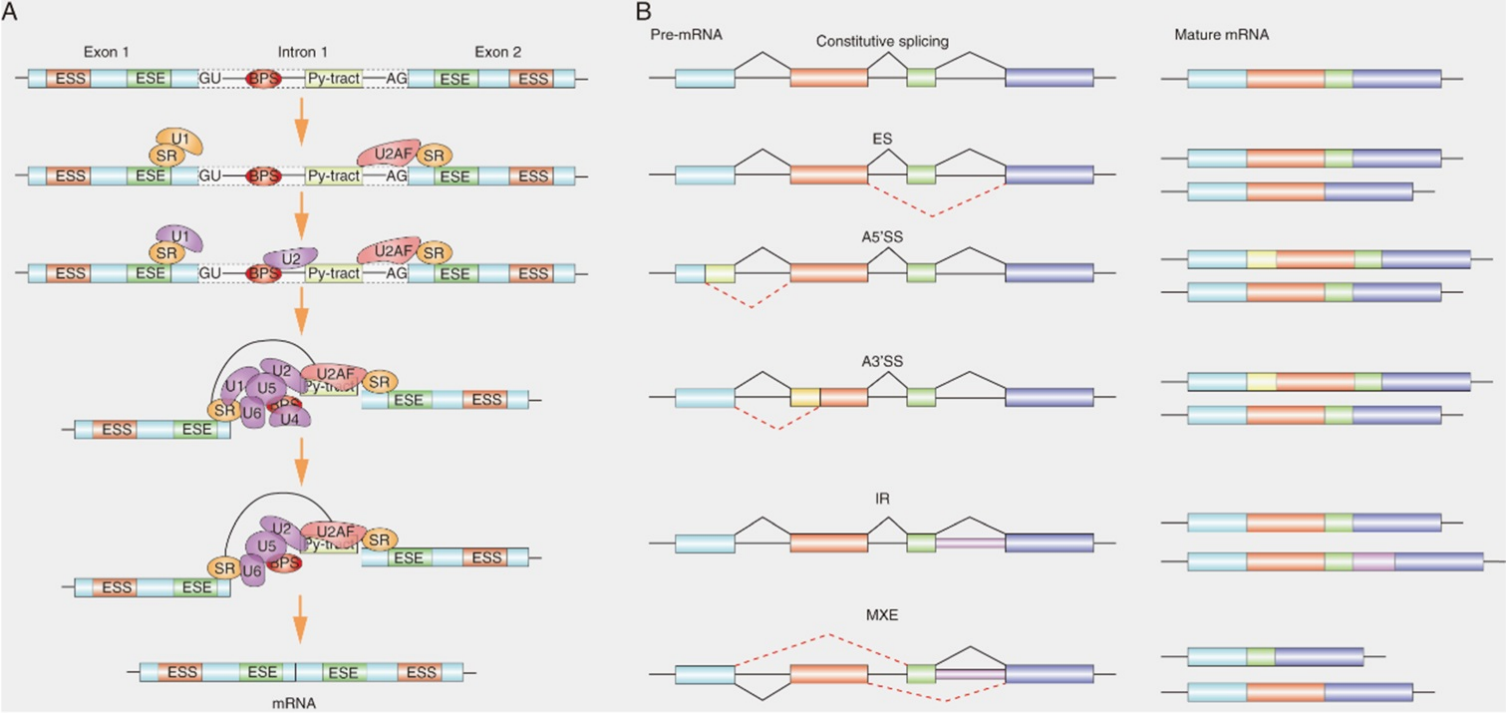
** Role of SRs in alternative splicing. (A)** Graphical representation of the stepwise assembly of spliceosomal complexes. SRs bound to ESE (exonic splicing enhancer) elements recruit U2AF35 to an upstream 3' ss (splicing site) and U1 to the downstream 5' ss. SRs and U2AF2 bind, respectively, to the branch point site (BPS) and the polypyrimidine tract (Py-tract).Next, SRs recruit U2 to the BPS of pre-mRNA and then SRs form a bridge complex across two splicing sites by binding to U2AF at 3' ss and U1 at 5' binding site. Recruitment of the preassembled U4/U6/U5 tri-snRNP. Finally, major conformational changes, including release of snRNP, lead to spliceosome activation and formation of the mature mRNA. **(B)** Schematic depicting constitutive splicing and five examples of alternative splicing patterns are exons skipping (ES), alternative 5' splice sites (A5' SS), alternative 3' splice sites (A3'SS), intron retention (IR) and mutually exclusive exons (MXE). Shown on the right are the mature mRNA transcripts derived from each alternative splicing event.

SRs commonly designate a family of RNA binding proteins that share common RNA binding motifs 15. The unique RS domain is an important structure that distinguishes SRs from other RNA-binding proteins. As the ubiquitous splicing factors, SRs play an important role in both constitutive and alternative splicing functions. In the splicing reaction, the SRs bind to exonic splicing enhancers (ESE), recruit small nuclear ribonucleoprotein (snRNP) and its cofactors to the splicing sites 27, Next, SRs facilitate the U2 snRNA binding to the branch point region of pre-mRNA to form complex A, and then they also bridge the communication between these initial splice site recognition events in the pre-spliceosome and the mature spliceosome to form a bridge complex across the splicing sites by binding to U2AF and U1 70K, thereby promoting the recruitment of U4/U5/U6 snRNP to form complex B 28 (Fig. 2). In addition, the locations of SRs in pre-mRNA sites affect the splicing outcome. For example, exon-combined SRs act as enhancers, but intron-combined SRs may as suppressors 29. Therefore, the location of SRs-pre-mRNA interactions affect the selection of splicing sites and the assembly of spliceosome 30.

#### Function of SRs in transcription

**Figure 3:**
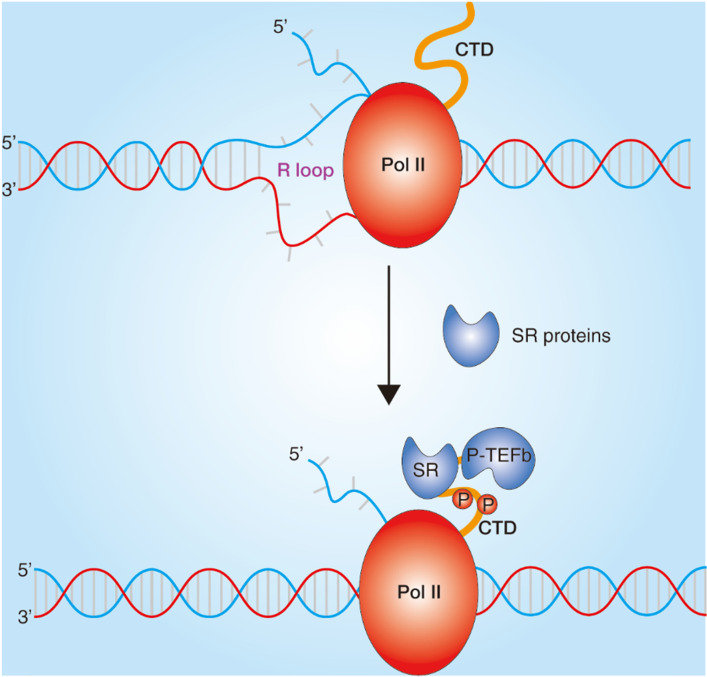
** Role of SRs in transcription.** Transcriptional regulation in chromatin. SRs bound to nascent RNA and impede the formation of R-loop and dynamically bridge P-TEFb to Pol II. Transcription elongation are regulated by Pol II phosphorylation in C-terminal repeat domain (CTD) and SRs interactions.

A large number of studies have found that SRs play a pivotal role in almost all processes of RNA metabolism. In addition to regulating RNA splicing, SRs also plays an important role in regulating transcription (Fig. 3) 31. SRs are concentrated in nuclear speckles which are small subnuclear membraneless organelles or structures, also called the splicing factor compartments and are from there recruited to nascent transcripts transcribed by RNA polymerase II (Pol II) 32. The interactions of SR-related proteins with the C-terminal domain of Pol II have been reported 33, and the SRs were also identified in the Pol II complex, which elucidates that SRs may contribute to transcriptional elongation 34. Further studies show that SRSF2 could dynamically bridge P-TEFb to Pol II and thereby further catalyzes Pol II phosphorylation in C-terminal domain to promote transcriptional elongation in genes 35. In addition, the co-transcriptional complex of SRs have been demonstrated to prevent the formation of R loops because of hybridization of the synthesized pre-mRNA to the complementary strand of the DNA template and thus to contribute to genome stability 14.

#### Function of SRs in the splicing of transcripts containing N6-methyladenosine (m6A) modifications

**Figure 4:**
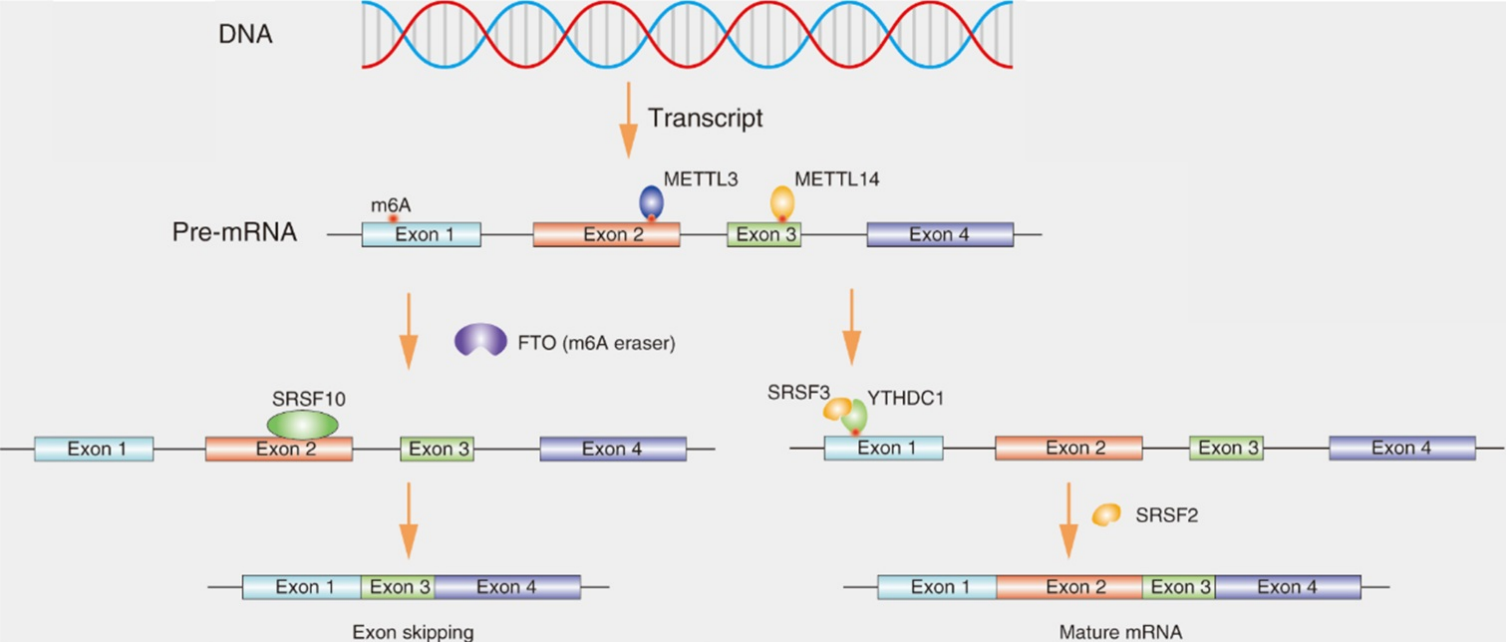
** SRs affects the splicing of transcripts containing N6-methyladenosine (m6A) modifications.** m6A methylation of RNA is catalyzed by the methyltransferase METTL3 and METTL14. YTHDC1 could effectively binds to m6A site and recruit SRSF3 to pre-mRNA, leading to exon inclusion. If fat mass and obesity-associated (FTO) demethylates the m6A site, as a result, SRSF10 instead of SRSF2 binds to pre-mRNA, leading to exon skipping.

N6-methyladenosine (m6A) is the most prevalent internal RNA modification, especially within eukaryotic messenger RNAs (mRNAs), affecting various aspects of biological processes including RNA splicing (Fig. 4) 36. m6A modifications are catalyzed by RNA methyltransferases, such as METTL3, METTL14 and WTAP (writers). The modifications are removed by the demethylases fat mass and obesity associated protein (FTO) and ALKB homolog 5 (ALKBH5) (erasers). The modifications are recognized by m6A-binding proteins, such as YTH domain-containing proteins and IGF2BPs (readers) 37. Disruption of the dynamic of m6A can affect the levels of many RNAs, because the methylation-related molecules are located in nuclear speckles and interact with splicing factors which may be involved in modulating splicing 38.Recent studies indicate that the downregulation of ALKBH5 can affect the distribution of splicing factors, such as SRSF1, SRSF2 and SRPK1 39. FTO could mediate splicing of RUNX1 through recruiting SRSF2 40. Xiao et al. reported that YTHDC1 promotes exon inclusion in targeted mRNAs through recruiting pre-mRNA splicing factor SRSF3 while blocking SRSF10 bound to the region near the m6A sites, ultimately resulting in exon skipping owing to the failure of m6A modification 41. Feng Z et al. also showed that METTL3 knockdown facilitates the formation of MyD88 isoform through regulating splicing 42.

## Role of SRs Family in Cancer

**Figure 5:**
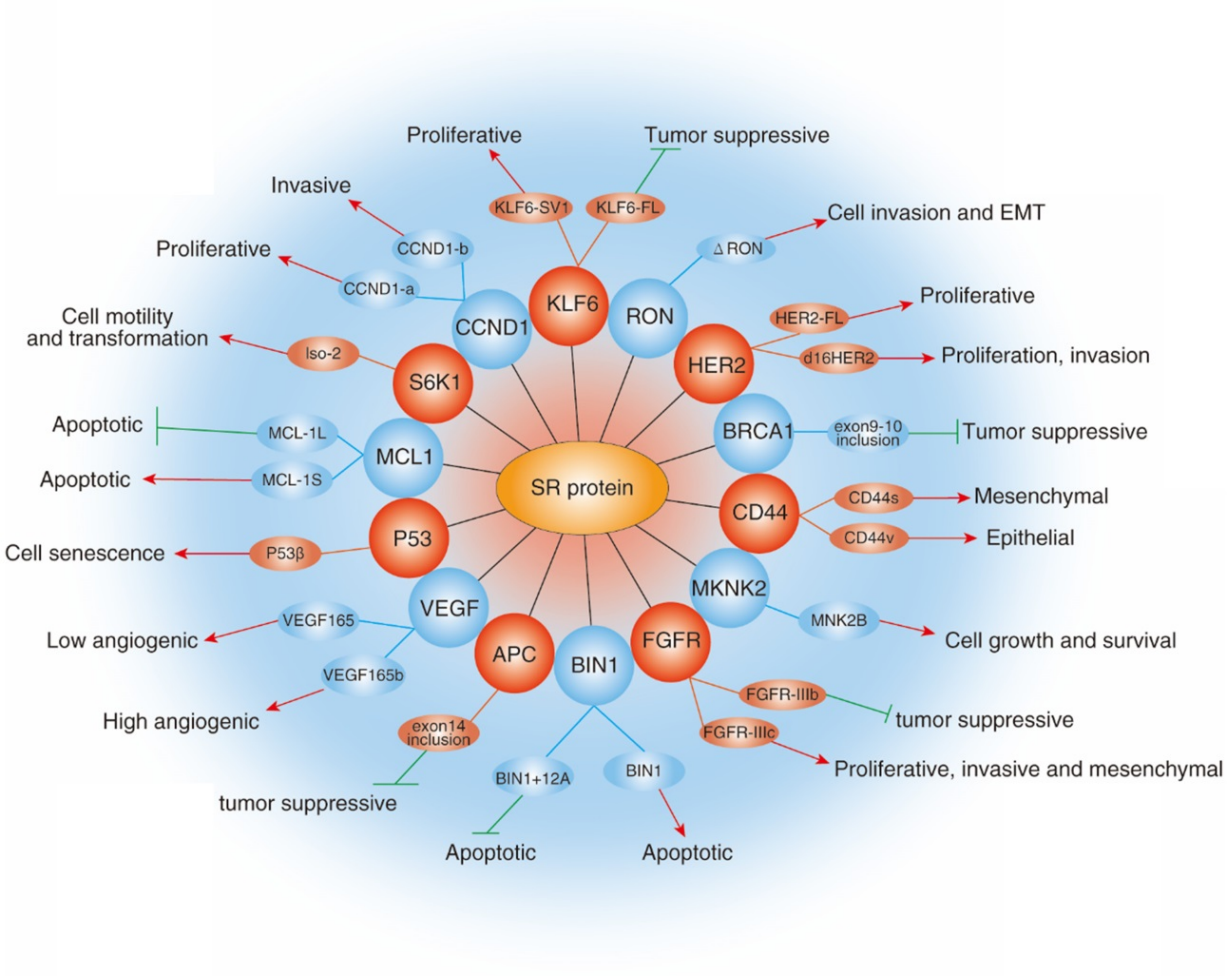
** Role of SRs family in cancer.** The diagram shows selected examples of genes with cancer-related alternatively spliced isoforms that due to the activity of specific SRs. Red arrowheads represent promotion and green lines represent inhibition.

Almost all tumors have complex pathogenesis with the participation of multiple factors. The above-mentioned studies provide enough evidence to support the correlation between SRs and cancers. Because SRs are the master regulator of pre-mRNA splicing, dysregulation of SRs greatly damage DNA stability and normal expression pattern of protein, and then result in aberrant biological function, which indicates a critical role for SRs in cancer. For example, Li et al found that depletion of SRSF1 *in vivo* could induce DNA double-strand break and gross DNA recombination 14. Similarly, Xiao et al also showed that loss of SRSF2 in mouse embryo fibroblasts induced double strand break and G2/M cell cycle arrest via regulating at least in part the p53 hyperphosphorylation and hyperacetylation 43. Indeed, a series of experiments have demonstrated that SRs are aberrantly expressed in human cancers, participating in tumorigenesis and cancer metastasis (Fig. 5).

### Abnormal expression of SRs and cancer

Growing evidence establish the relationship between the dysregulated expression of SRs and tumorigenesis which mainly owing to changing in the alternative splicing patterns of key genes. It has been reported that copy number of SRs are dramatically increased in various cancers, which are related to the high expression of SRs in cancers 18. For example, SRSF6 has been found to be massively amplified in lung, colon and breast cancers 44. Using both discovery and validation patient cohorts, Jiang L et al. reported that SRSF1 DNA copy number gain and mRNA over-expression were strongly associated with poor survival, and they further demonstrated that SRSF1 is important for tumorigenesis of SCLC (small cell lung cancer) and may play a key role in DNA repair and chemo-sensitivity both *in vitro* and *in vivo*. Moreover, SRSF1 promotes SCLC growth and survival by regulating PI3K/AKT and MEK/ERK signaling pathways, which are well-known as oncogenic signaling pathways 21. Increased expression of SRs usually correlate with cancer progression, as in malignant ovarian tissue, the expression of SRSF1, SRSF2 and SRSF3 were significantly upregulated relative to normal tissue 45. Park WC et al. found that the expression of SRSF7 was higher in gastric cancer than normal gastric tissue 46. Song et al also observed that the level of SRSF7 was upregulated in NSCLC tissues, meanwhile, the expression level of SRSF7 was negatively correlated with miR-374b-5p and positively correlated with MALAT1. Overexpression of SRSF7 could reverse the effects of MALAT1 knockdown on proliferation, apoptosis, and invasion in NSCLC cells 47. In addition, SRSF1 was found to be upregulated in colon, kidney, lung, liver, pancreas and breast tumour 18, 20, 48, 49. Kim et al reported that the expression level of SRSF5 was markedly up-regulated in lung cancer samples compared with normal lung tissue samples and SRSF5 showed the higher detection accuracy (89%) than carcinoembryonic antigen(74%)(CEA, a commonly marker of non-small cells lung cancer (NSCLC) 50. Yang et al also discovered that SRSF5 is overexpressed in oral squamous cell carcinoma tissues and cells. The expression of SRSF5 is controlled by an autoregulation mechanism 51. Fu et al demonstrated that SRSF9 is frequently overexpressed in many types of tumors through immunohistochemistry staining, such as glioblastoma, colon adenocarcinoma, squamous cell lung carcinoma and malignant melanoma 52. Other SRs, such as SRSF3, SRSF5 and SRSF6 also presented a higher expression level in colorectal cancer than normal tissue 46.

Abnormal expression of SRs often causes changes of cancer-related processes, such as epithelial-mesenchymal transition, metastasis and angiogenesis. Kong et al identified that SRSF6 promoted the EMT and metastasis in colorectal cancer, and found that the knockdown of SRSF6 significantly increased the expression of E-cadherin as well as reduced the expression of Snail, Vimentin, Fibronectin and MMP9 in SW620 cells 53. In addition, Wan et al also found that SRSF6 could promote invasion and metastasis in colorectal cancer by inhibiting ZO-1 exon23 inclusion, which known to play an inhibitory role in colorectal cancer progression 54. Recently, Zhou et al reported that SRSF1 is increased in glioma tissues and cell lines. Moreover, Further studies revealed that SRSF1-mediated splicing of the MYO1B increased the potential of proliferation, and metastasis in glioma cells by regulating the PDK1/AKT and PAK/LIMK pathway 55. Angiogenesis is modulated by the balance of pro-angiogenic VEGF165 and anti-angiogenic VEGF165b splicing isoforms. Manetti et al demonstrated that SRSF6 could involve in distal splice-site selection as it bound around the distal splice-site and was up-regulated by TGFb, resulting in increased the expression of VEGF165b and inhibition of angiogenesis 56.

### Splicing function of SRs and cancer

Most genes undergo alternative splicing, and the isoforms produced by alternative splicing are closely related to the progression and metastasis of cancers 57, such as pro-oncogenic and antiapoptotic splicing isoforms of MDM2, BCL(X), and VEGF genes 58. In tumor cells, SRs cause abnormal splicing of many tumor-related genes, which affect cell proliferation, apoptosis and migration, and thereby promotes tumorigenesis and cancer development 59, 60. Karni R et al. found that upregulated SRSF1 protein increased the splice isoform of BIN1 (bridging integrator-1). The resulting BIN1 isoforms cannot bind to the oncoprotein MYC, causing the BIN1 protein to lack tumor-suppressor activity 18. A series of studies found that dysregulated expression of SRSF1 protein can also alter alternative splicing of many other tumor-related genes. For example, in prostate cancer, SRSF1 promotes the increase of oncogenic cyclin D1b splice isoform 61. In lung cancer, SRSF1 promotes the expression of the anti-apoptotic caspase 9b splice isoform 62. In breast cancer, SRSF1 regulates the alternative splicing of the pro-apoptotic gene BIM, which increases the expression of splice variant lacking the BH3 domain, thereby inhibiting apoptosis 63. In addition, the alternative splicing regulated by SRSF1 in tumor cells also includes the proto-oncogenes MYBL2 (B-myb), MYB (C-myb) 64, the tumor suppressor gene BRCA1 65, and the apoptosis-related gene Mcl1 66, Vascular endothelial growth factor VEGF 67, cell migration-related gene MST1R (Ron) 49, etc. In addition, Kurokawa K et al. found that SRSF3 can regulate apoptosis by affecting alternative splicing of HIPK2 (homeodomain-interacting protein kinase-2) and produced a HIPK2 Δe8 isoform in colon cancer cells 68. Liu et al found that compared with control, the expression of SRSF4 was obviously decreased in Acute Myeloid Leukemia (AML) patients. Moreover, an important correlation was observed between SRSF4 and the caspase-8 splicing isoforms in AML patients 19. Shi J et al. reported that SRSF2 involved in the regulation of alternative splicing of the tumor suppressor gene KLF6 (Krüppel-like factor 6) and promoted the production of splice isoform containing KLF6 exon 1a. The isoform of KLF6 which cannot inhibit tumorigenesis due to the absence of the DNA binding domain 69. SRSF6 can regulate the alternative splicing of tumor-related genes, such as the insulin receptor INSR, the kinase Mnk2 (MKNK2) and the tumor suppressor gene DLG-1, thereby mediating tumorigenesis 70-72. Zhou X et al. showed that SRSF10 was up-regulated in colon cancer samples, and inhibition of SRSF10 could significantly inhibit the proliferation and tumorigenesis in colon cancer cells by regulating the expression of Bcl-2-associated transcription factor 1 (BCLAF1) isoform containing BCLAF1 exon5a 73. Recently, Liu et al showed that SRSF10, was upregulated by human papillomavirus oncoproteins E6 and E7 through E2F1 pathway, and promoted the tumorigenesis of cervix. Further investigation that SRSF10 could promote the expression of CD47 to inhibit macrophage phagocytosis by promoting nuclear factor-κB activation by regulating the splicing of interleukin-1 receptor accessory protein exon 13.

Recent genome-wide analyses of cancer have revealed globally abnormal splicing profiles in mature mRNA 74, 75. The molecular mechanisms of aberrant splicing in cancers may be mutations in SRs, such as SRSF2 76. SRSF2 mutation is found in 40% -50% of patients in chronic myelomonocytic leukaemia 77. SRSF2 missense mutation are highly concentrate on at residue P95 or to a lesser extent at the surrounding residues (G93 to P107) 78. SRSF2 canonically binds to C- and G-rich exonic splicing enhancers (ESEs) motifs to promote splicing 79. The mutant SRSF2 preferentially recognizes C-rich CCNG ESE motif versus G-rich GGNG ESE motif and results in alternatively splicing of many mRNAs 80. For example, Papaemmanuil et al reported that SRSF2 mutations altered the splicing of EZH2 mRNA that induced an in-frame stop codon in myeloid neoplasms. The abnormal EZH2 mRNA induces nonsense‑mediated decay (NMD) and leads to the downregulation of EZH2 protein and histone H3 lysine 27 trimethylation levels 81.

### Translation function of SRs and cancer

We mentioned above that the nuclear shuttle SR proteins can directly regulate mRNA translation 82. Sanford JR et al. reported that SRSF1 could stimulate translation both *in vitro* using a HeLa cell-free translation system and in xenopus oocytes system 83. As an adaptor protein, SRSF1 recruits mTORC1 or PP2A to its bound mRNA, and increases the phosphorylation level of 4E-BP1. Phosphorylated 4E-BP1 no longer binds to the initiation factor eIF4E, which promotes mRNA translation initiation 84. Karni R et al. have found that overexpression of SRSF1 could activate the mTORC1 signaling pathway, resulting in increased phosphorylation of its downstream target proteins S6K and 4E-BP1, and that the tumorigenesis of SRSF1 can be inhibited by the mTOR-specific inhibitor rapamycin 85. Moreover, SRSF1 can activate the Ras-MAPK signaling pathway and increase cell transformation by increasing B-Raf expression 86. Maslon MM et al. also performed a high-throughput deep sequencing analysis of polysomal fractions in cells overexpressing SRSF1 and identified approximately 1,500 mRNAs that were translational targets of SRSF1. These include mRNAs encoding proteins involved in cell cycle regulation, such as spindle, kinetochore and M phase proteins, which are essential for accurate chromosome segregation 87. These findings suggest that the regulation of translational functions of the SRSF1 protein promotes, to a certain extent, SRSF1 protein-mediated cell transformation and tumorigenesis. Other shuttling SR proteins like SRSF7 and SRSF3 also have the function of regulating translation 88, 89. For example, SRSF3 was reported to involve in the regulation of IRES (internal ribosomal entry site)-mediated translation initiation. Further investigation found that SRSF3 interacted with the RNA binding protein PCBP2 that bound to IRES sequences in the genomic RNAs of viruses and was necessary for viral translation. 88.

### Post-translational modification of SRs and cancer

At least five types of posttranscriptional modifications are known in SRs, including methylation, acetylation, small ubiquitin-related modifier (SUMO), ubiquitination and phosphorylation 22. There has been considerable interest in modulating splicing by modifying post-translational modifications (PTM) required for spliceosome function, and known that PTMs of a variety of splicing factors regulate spliceosome formation and splicing catalysis 90. Arginine methylation has been detected on many RNA binding proteins, such as hnRNPs and SRs 91. Arginine methylation in SRs was first reported on Npl3p in budding yeast 92. In addition, Sinha R et al. reported that three methylated arginine residues were identified between the two RRMs in SRSF1, which appeared to play an important role in promoting nuclear import of the SRs. Further studies showed that arginine control the subcellular localization and the positive charge state. Mutations or block methylation and remove the positive charge lead to the cytoplasmic accumulation of SRSF1 93. Botti et al also showed that arginine methylation in SRSF5 is important for its shuttling capacity and mRNA export 94. Ubiquitin proteasome pathway-mediated protein degradation is an important mechanism by which the cell regulates intracellular protein levels and functions. Vaishali et al reported that ubiquitination can regulate the expression of SRSF1 in normal and T cells 95. In addition, Osman et al showed that SRSF5 protein is decreased drastically during cell differentiation by proteasome-mediated proteolysis 96. Chen et al also showed that mutually exclusive ubiquitylation and acetylation of SRSF5 control tumor growth by regulating the alternative splicing of CCAR1 to generate the CCAR1S isoform, which promote tumor growth by promoting glucose consumption and acetyl-CoA production 97. In addition, reversible phosphorylation in the splicing reaction was necessary. Phosphorylation of SRs modulates the shuttling of SRs to modulate splicing. Phosphorylation of SRs is necessary for spliceosome assembly, but dephosphorylation of SRs allows splicing catalysis to occur and initiates nuclear export of SRs 28, 98, 99. Phosphorylation of the RS repeat domain could release the stable α-helical structure to form the "arginine claw" structures which is recognized by a SRs specific transportin (TRN-SR), thereby trafficking the SRs to nucleus where it involves in splicing 100. Serine-arginine protein kinase (SRPK) can phosphorylate Arg-Ser dipeptide repeats of SRs and promote nuclear import 101. Abnormal expression of SRPK1 damages the biological function of SRs, leading to aberrant splicing of pre-mRNA, which can contribute to the progression of cancers 102. Matos et al reported that SRPK1 could regulate the alternative splicing of Rac1b that enhanced cell cycle progression via NF-kB signaling 103. SRPK1 contributes to the splicing of Rac1b by inducing the phosphorylation of SRSF1 to further promote its entry into the nucleus 104. Small ubiquitin-related modifier (SUMO) is a reversible and transient posttranslational protein modifier and involved in diverse biological processes, such as subcellular partitioning, transcription regulation, DNA damage repair, stress response and chromatin remodeling. Federico et al showed that the SRSF1 was a modulator of the sumoylation pathway. SRSF1 can enhance sumoylation of specific substrates by interacting with Ubc9. SRSF1 also can regulate SUMO E3 ligase protein inhibitor of activated STAT-1(PIAS1)-induced overall protein sumoylation by interacting with the PIAS1 105. In recent years, proteomic analysis has also shown extensive lysine acetylation in SRs, which may be an important nonhistone substrates recognized by histone acetyltransferases (HATs) in cells 106. Edmond V et al. found that the acetyltransferase Tip60 acetylated SRSF2 on its lysine 52 residue inside the RNA recognition motif, thereby promoted its proteasomal degradation, while the deacetylase HDAC6 countered this acetylation and acted as a positive regulator of SRSF2 protein level 107.

### Other functions of SRs in cancer

SRs regulate RNA metabolism at multiple levels. In addition to participating in the regulation of RNA splicing and translation efficiency, SRSF1 protein also plays a role in the nuclear export and stability of RNA. These functions may be involved in SRSF1-mediated tumorigenesis. For example, SRSF1 is highly expressed in non-small cell lung cancer. Down-regulating SRSF1 in non-small cell lung cancer cell lines can promote the occurrence of apoptosis, which is related to the down-regulation of anti-apoptotic protein survivin. Mechanism shows that SRSF1 specifically binds to survivin mRNA and regulates its expression at multiple levels. It not only relies on the mTOR signaling pathway to promote translation of survivin mRNA, but also stabilizes its mRNA level. The analysis of clinical tumor samples further confirms that there is a positive correlation between SRSF1 and survivin in non-small cell lung cancer. These results provide new data on the mTORC1- and survivin-dependent mechanisms of SRSF1-related carcinogenic potential, and suggest that the expression of SRSF1 and survivin is involved in NSCLC progression 108. SRSF1 is also up-regulated in breast cancer. Overexpression of SRSF1 promotes cell transformation of breast epithelial cells both *in vitro* SRSF1-overexpressing MCF-10A cells and *in vivo* SRSF1-overexpressing COMMA-1D transplantation models, and promotes proliferation and inhibits apoptosis in three-dimensional culture cells, forming more acinar than normal cells 63. Overexpression of SRSF1 in mammary epithelial cells not only regulates the alternative splicing of BIM and BIN1 to inhibit apoptosis, but also cooperates with the oncogene MYC to promote cell transformation 63. Subsequent studies have further confirmed that MYC regulates its downstream splicing activity and tumorigenesis by promoting SRSF1 transcriptional activation 63.

## Role of SRs in Antitumor Therapies

Because SRs play an important role in RNA metabolism, during the past decades, increasing strategies have been made to target the spliceosome for antitumor therapeutic approaches 109, 110. As described above, it has been extensively reported that widespread changes in RNA splicing in cancer samples compared with normal samples, such as known pro-oncogenic and antiapoptotic isoforms of genes, including TP53, BCL (X), and VEGF 58, 111, 112. Guyot et al. reported that anti-angiogenic forms of VEGF isoform VEGF165b can weaken the therapeutic effects of the anti-VEGF antibodies (bevacizumab/Avastin) in different tumor cell lines. 113. These findings showed that regulating pre-RNA splicing of specific gene isoforms may serve as a new therapeutic strategy for cancer. Recently, a series of studies unveiled diverse compounds that targeted the spliceosome. Among the first compounds known to alter splicing are natural products (spliceostatin A, pladienolide B, and herboxidiene) that bind the SF3B complex 114, 115, and their derivatives including E7107 and H3B-8800 116, which physically bind to the SF3B complex and inhibit pre-mRNA splicing at an early step in spliceosome assembly and thereby block splicing. In addition, Lee SC et al. showed that mutated SRSF2 are more sensitive to E7107 in comparison to cells bearing wild-type SRSF2 in leukemia, suggesting that cancers induced by spliceosome mutated factors was more sensitive to these drugs 117.

Chemoresistance and radioresistance are the major cause of cancer treatment failure. Splicing dysregulation plays key roles in tumorigenesis and the involvement of SRs in resistance of cancer to chemotherapy and radiotherapy remains elusive 118. Recently, The expression level of SRSF1 was found increased in irradiation treated lung cancer cells, whereas interference of SRSF1 sensitized lung cancer cells to irradiation by regulating various cancer-related splicing, such as the splicing of PTEN-like mitochondrial phosphatase (PTPMT1) 118. Maude et al reported that SRSF4, but not SRSF6 played an important role in anti-cancer drug cisplatin affected splicing, and knockdown of SRSF4 expression inhibited lots of the splicing alterations and cell death induced by cisplatin 119.

Alternative splicing regulation is also a key target for anti-tumor treatment. SRs are phosphorylated by SRPK1 and SRPK2 (SRs kinases), DNA topoisomerase I and U4/U6 small ribonucleoprotein PRP4 120. Through regulation of SRs phosphorylation, small molecule inhibitors that target kinases involved in alternative splicing are promising candidates for drugs 121. Pilch B et al. found that NB-506, an inhibitor of topoisomerase I, could inhibit the phosphorylation of SRSF1 and modulate gene expression by controlling splicing 122. In addition, Wu F et al. reported that antibody targeting SRPK1, a key regulator of SRs phosphorylation, significantly inhibited the growth, migration and invasion of non-small cell lung cancer 123. PRP4 belongs to the serine/threonine protein kinase family, plays an important role in cell mitosis and splicing. PRP4 kinase has been identified as a potential therapeutic target in multiple tumors. For example, Duan et al found that inhibition of PRP4 activity by shRNAs could reverse paclitaxel resistance 5-10-fold in human ovarian cancer 124. Jouhua et al also showed that inhibition of PRP4 could enhance paclitaxel activity in breast cancer cells 125.

In another example, symmetric arginine dimethylation (SDMA), catalyzed by PRMT5, is required for spliceosome assembly 126, 127. Some studies shown that compounds inhibition of PRMT5 results in splicing inhibition and anticancer effects in a number of cancer types 128, 129. Recent studies positioned RNA methylation as a major pathway in modulating the development of tumor due to its function in post-transcriptional regulation including alternative splicing. It is of great significance to identify chemical inhibitors targeting m6A modification-related enzymes, including METTL3, METTL14, FTO and ALKBH5, etc 130.

## Conclusions and Perspectives

Alternative splicing is a vital step in gene expression and generates transcriptome diversity in eukaryotic cells. As the key regulators of cellular biology, SRs are extensively participated in regulating from RNA stability to RNA splicing, and even RNA modification(m6A), export and translation 14, 84, 131. Moreover, the expression of most SRs in various tumor types is up-regulated, and therefore SRs promotes tumorigenesis as proto-oncogenes. SRs mediate tumorigenesis mainly by regulating alternative splicing of tumor-related genes, providing a large number of targets for tumor prevention and treatment. Since most of the current research is focused on a single SR protein, this also leads to some interesting problems that have not been well explained. For example, do SRs regulated targets have something in common? are these targets regulated by many SR proteins or specifically by one? is the regulatory mechanism similar for all these targets? At present, it is known that antisense oligonucleotides or small molecule drugs can be used to regulate some alternative splicing processes that mediate tumorigenesis 132. These may be one of the measures to achieve tumor treatment by targeting SRs in tumorigenesis. However, due to relatively few animal models system for knock-out/knock-in SRs, the regulation of SRs is still incomplete, and more research is needed in this area to further determine the functions and mechanisms of different SRs in tumorigenesis, which will provide more abundant research ideas and treatment strategies for tumor treatment. In addition, with the broad application of bioinformatics and high-throughput sequencing technology in research, increasing numbers mutations of SRs have been found in tumors. What specific role do these mutations play in the progression of tumors? And whether repairing these mutations can be used as a potential treatment strategy is still unknown. Of course, I believe that with the advancement of research methods, more and more mysteries about SRs will be unveiled.

## Figures and Tables

**Table 1:** Dysregulated SRs' expression in cancer

SRs	Cancer Type	Target Gene	Effects on Cancer Progression	References
SRSF1	Breast, Lung, and Colon cancer	Cyclin D1, CASP9, BIN1, Mnk2 and RON1	Cell proliferation, invasion, migration and apoptosis	49, 63, 133-135
SRSF2	Lung and renal cancer	Caspase-8, BCL-x, MCL-1, Survivin and BIM	Apoptosis	136, 137
SRSF3	Breast cancer	MCL-1	Apoptosis	66
SRSF4	Acute Myeloid Leukemia	Caspase-8	Apoptosis	19
SRSF5	Breast cancer	MCL-1	Apoptosis	66
SRSF6	Melanoma	BIM	Apoptosis	138
SRSF7	Colon and lung cancer	Fas	Apoptosis	47
SRSF9	Colon cancer	b-catenin	Proliferation	52
SRSF10	Colon cancer	BCLAF1	Apoptosis	73

## Abbreviations

SRs/SRSF: serine/arginine-rich splicing factors
AS: Alternative splicing
m6A: N6-methyladenosine
RBP: RNA-binding protein
ESE: exonic splicing enhancer
ISE: intronic splicing enhancer
ESS: exonic splicing silencer
ISS: intronic silencer
RRM: RNA recognition
hnRNP: heterogeneous nuclear ribonucleoparticle protein
snRNP: small nuclear ribonucleoprotein
P-TEFb: The positive transcription elongation factor b
METTL3: Methyltransferase-Like Protein 3
METTL14: Methyltransferase-Like Protein 14
WTAP: Wilms Tumor 1 Associated Protein
FTO: Fat Mass and Obesity-Associated Protein
ALKBH5: Alpha-Ketoglutarate-Dependent Dioxygenase AlkB Homolog 5
YTHDF: YTH Domain-Containing Family Protein 1
IGF2BP: Insulin Like Growth Factor 2 MRNA Binding Protein
RUNX1: RUNX Family Transcription Factor 1
YTHDC1: YTH Domain-Containing Protein 1
SCLC: small cell lung cancer
BIN1: Bridging Integrator 1
MYC: MYC proto-oncogene
BIM: Bcl-2-Like Protein 11
MYBL2: MYB Proto-Oncogene Like 2
MYB: MYB Proto-Oncogene
BRCA1: Breast Cancer Type 1 Susceptibility Protein
Mcl1: Myeloid Cell Leukemia Sequence 1
VEGF: Vascular Endothelial Growth Factor
MST1R: Macrophage Stimulating 1 Receptor
HIPK2: Homeodomain Interacting Protein Kinase 2
KLF6: Kruppel Like Factor 6
INSR: Insulin Receptor
Mnk2: MAPK Interacting Serine/Threonine Kinase 2
DLG-1: Discs Large MAGUK Scaffold Protein 1
MTORC1: Mechanistic Target of Rapamycin Kinase Complex 1
PP2A: Protein Phosphatase 2 Phosphatase Activator
S6K: Ribosomal Protein S6 Kinase B1
4E-BP1: Eukaryotic Translation Initiation Factor 4E Binding Protein 1
MAPK: Mitogen-Activated Protein Kinase
B-Raf: B-Raf Proto-Oncogene Serine/Threonine Kinase
PTM: post-translational modifications
HATs: histone acetyltransferases
HDAC6: Histone Deacetylase 6
SF3B: Splicing Factor 3b Subunit
SRPK1: Serine/Arginine-Rich Protein-Specific Kinase 1
SRPK2: Serine/Arginine-Rich Protein-Specific Kinase 2
PRP4: Pre-MRNA Processing Factor 4
SDMA: symmetric arginine demethylation
RMT5: Protein Arginine Methyltransferase 5
